# Preparation of a carboxymethyl β-cyclodextrin polymer and its rapid adsorption performance for basic fuchsin†

**DOI:** 10.1039/c9ra10797e

**Published:** 2020-06-02

**Authors:** Hongyu Pu, Peixiao Tang, Ludan Zhao, Qiaomei Sun, Yuanming Zhai, Zhiqiang Li, Na Gan, Yuanyuan Liu, Xiuyun Ren, Hui Li

**Affiliations:** School of Chemical Engineering, Sichuan University Chengdu 610065 P. R. China lihuilab@sina.com tangpeixiao@126.com +86 028 85401207 +86 028 85405220; Analytical & Testing Center, Sichuan University Chengdu 610064 P. R. China; China Tobacco Yunnan Industrial Co., LTD. Kunming 650231 P. R. China

## Abstract

The presence of dyes in a water system has potential adverse effects on the ecological environment. The conventional cyclodextrin (CD) polymer only has CD cavities as adsorption sites and exhibits slow adsorption for dye removal. In this study, we designed a novel carboxymethyl β-CD polymer (β-CDP-COOH). The structural properties of β-CDP-COOH were characterized as an irregular cross-linked polymer with negative surface charge, and the introduction of carboxymethyl groups greatly enhanced the adsorption ability of the β-CD polymer to basic fuchsin (BF). The maximum removal efficiency of β-CDP-COOH (96%) could be achieved within 1 min, whereas that of conventional β-CD polymer (70%) was achieved after 50 min. The adsorption mechanism revealed that the adsorption behavior of β-CDP-COOH could be effectively fitted with the pseudo-second-order kinetic model and Langmuir isotherm. Both CD cavities and carboxymethyl groups were effective adsorption sites, so β-CDP-COOH had an advantage in adsorption capacity over the conventional β-CD polymer. This study indicated that β-CDP-COOH is a potential highly efficient adsorbent for the removal of cationic dye contaminants.

## Abstract 2

Introduction of carboxymethyl groups greatly accelerated the adsorption rate of a β-CD polymer, and the removal efficiency reached 96% within 1 minute.

## Graphical abstract

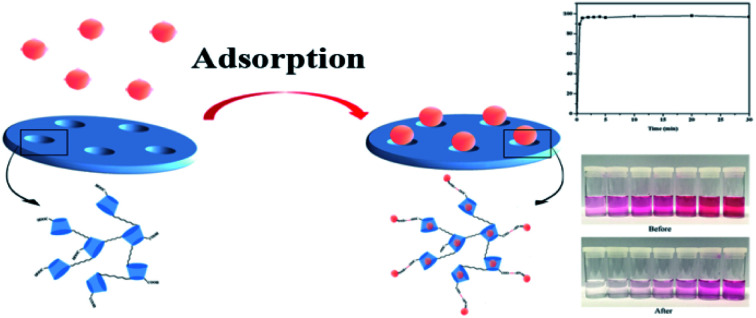

## 1. Introduction

**Fig. 1:**
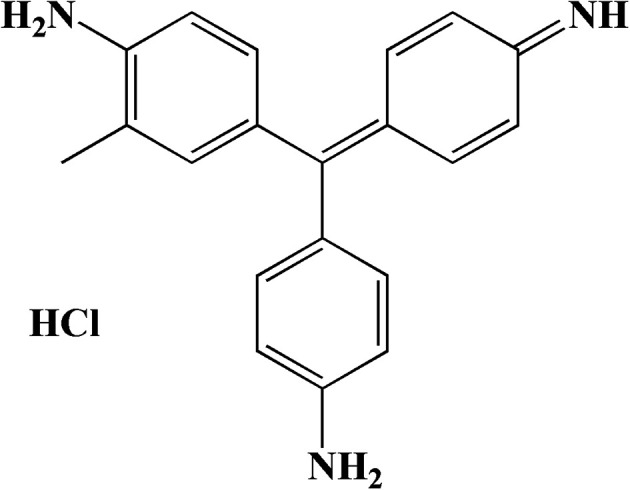
Structure of basic fuchsin.

Basic fuchsin (BF; Fig. 1) is an extensively used organic cationic dye in textiles, leather materials, paper, and biology.^1^ However, given its high tinctorial value,^2^ BF can cause non-negligible color pollution, reduce the transparency of water, and affect the growth of aquatic organisms even at low concentrations.^3^ The complex aromatic structure of BF contributes to its toxicity, including skin contact irritation, organ and nervous system damage, carcinogenic and mutagenic effects,^4^ and difficult decomposition.^5^ These effects can harm the environment through biological accumulation in a material cycle.^6^ Therefore, finding a facile and effective way to treat BF wastewater is of great significance to the environment and human health.

Based on the potential environmental pollution caused by BF, the research on removal of BF has become the focus of extensive attention.^5^ The use of chemical coagulation,^7^ biotechnological methods,^8^ electrochemical methods,^9^ photocatalytic degradation,^10^ and new solid material adsorption is frequently reported.^1–5,11–18^ Among them, solid material adsorption has attracted increasing attention due to its advantages of high efficiency, easy separation, and economy.^19^ For example, anionic polyacrylamide/graphene oxide aerogels are highly effective adsorbents with a maximum adsorption capacity of 1034 mg g^−1^ and equilibrium time of 4200 min.^11^ Bottom ash is an inexpensive power plant waste material,^1^ and low-cost porous ceramic microspheres obtained from waste gangue have a maximum adsorption capacity of 24 mg g^−1^ and equilibrium time of 60 min (Table S1†).^13^ The common concern of researchers is the adsorption effect of materials, because it can address the slow adsorption rate and low adsorption capacity.

β-Cyclodextrin (β-CD) has remarkable ability in generating inclusion complexes with organic molecules in solution through host–guest interactions,^20,21^ and it has gained considerable attention in the field of environmental clean-up.^22,23^ The high density of hydroxyl groups inside and outside the β-CD cavity shows that these groups are active and easily functionalized.^18^ However, the removal of dyes is accompanied with slow adsorption and a long equilibrium time, because the only adsorption sites are CD cavities.^18^ BF can dissociate ammonium ions in aqueous solution and interact with carboxyl groups to form amine salts. Therefore, carboxyl groups may function as adsorption sites with high activity. However, in previous studies, carboxyl groups were introduced mainly through the cross-linking agents themselves or modification of carboxyl compounds such as carboxymethyl cellulose outside the polymer to increase the active sites. Limited studies have reported on the direct carboxylation of β-CD polymer, and no attention has been paid to the difference between β-CD polymer (β-CDP) and carboxymethyl-modified β-CD polymer in the removal of BF.

**Scheme 1:**
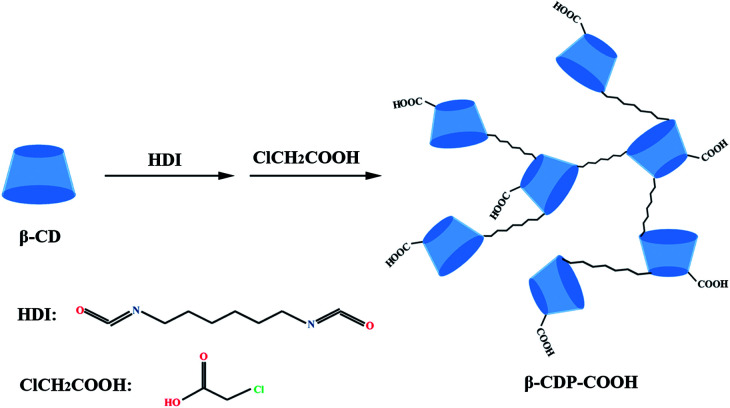
Preparation of the carboxymethyl β-CD polymer.

In this study, to increase the adsorption sites and improve the adsorption effect, a novel and efficient adsorbent (abbreviated β-CDP-COOH) was synthesized by β-CD polymerization and carboxymethylation (Scheme 1). The active adsorption sites and the negative electricity of the adsorbent were increased by the introduction of the carboxymethyl group, and the removal efficiency of BF in a water system was investigated. The adsorption kinetic, isotherm models and reuse of adsorbent were also evaluated. Meanwhile, the mechanism of BF scavenging by β-CDP-COOH was preliminarily investigated. Results showed that the introduction of carboxymethyl structure modification technology significantly improved the removal effect, and β-CDP-COOH has potential application value in removing BF wastewater.

## 2. Materials and methods

### 2.1 Materials

β-CD (purity ≥ 99%) and BF were obtained from Ke-Long Chemical Reagent Factory (Chengdu, China). Hexamethylene diisocyanate (HDI, purity ≥ 99%) and stannous octoate were obtained from Aladdin Industrial Corporation (Shanghai, China). Triple-distilled water was used throughout the experiment. The other reagents used were of analytical grade.

### 2.2 Synthesis of β-CDP and β-CDP-COOH

β-CDP was synthesized based on the reaction of isocyanate with the hydroxyl group.^24^ β-CD (1.0 g) and stannous octoate (one drop) were dissolved in 10 mL of DMF, and the mixture was added to a dried 250 mL three-neck round bottom flask. HDI (0.3 g) was dissolved in 5 mL of DMF and added to the mixture. The reaction was performed at 70 °C for 12 h under a nitrogen atmosphere. β-CDP precipitated out immediately after contact with chloroform. After resting for a few hours, the precipitate was collected by filtration and washed several times with triple-distilled water. The solid polymer was dried at 60 °C in vacuum.

β-CDP-COOH was prepared by modifying β-CDP. After 12 h of reaction between β-CD and HDI, NaOH (0.5 g) and monochloroacetic acid (0.167 g) were added to the three-necked flask, and the reaction continued at 50 °C for 5 h. After cooling to room temperature, the product was dried at 60 °C in vacuum. The resulting solid product was washed with triple-distilled water several times to remove any unreacted chemicals and then dried in vacuum.

### 2.3 Characterizations

Fourier transformed infrared (FTIR) spectra were recorded in solid state (KBr pellet method) for adsorbents and in liquid state for HDI by using an FTIR spectrometer (Thermo Fisher Scientific, USA), and the scanning range was 4000–400 cm^−1^. Powder X-ray diffraction (PXRD) patterns were obtained by X'Pert PRO diffractometer (PANalytical, Netherlands) with Cu Kα1 radiation in the scan range of 4°–40°, and the solid-state ^13^C NMR spectra were recorded in the solid state using a 600 MHz spectrometer (Bruker Avance, Germany) at 25 °C. Images were collected by a QUANTA 250 scanning electron microscope (FEI, USA) after the surface was coated with gold. The specific surface area of the adsorbents was calculated with the Brunauer–Emmett–Teller (BET) equation using a TriStar 3000 Surface Area and Pore Size Analyzer (HOSIC, UK). A 209F1 thermogravimetric analyzer (NETZSCH, Germany) with heating at 10 °C min^−1^ from 30 °C to 600 °C was used to perform thermogravimetric analysis (TGA) under a nitrogen atmosphere. The zeta potential of the polymers in water suspension was analyzed using a dynamic light scattering (DLS)-based Zeta PALS + BI-90Plus instrument (Brookhaven Instrument Co., USA) at 20 °C ± 1 °C.

### 2.4 Water regain analysis method

Water regain, an important property of materials for water treatment, can be used to investigate the hydrophilicity of absorbents.^25^ The adsorbents were dispersed in triple-distilled water for 1 h, and the wet adsorbents were collected and blotted by filter paper. The absorbents were weighed, and three parallel measurements were performed. Water regain was calculated by the following equation:

1 Water regain (%) = (*W*_w_ − *W*_d_)/*W*_d_ × 100%

where *W*_d_ (mg) and *W*_w_ (mg) respectively represent the mass of the adsorbents in the dry and wet states.

### 2.5 Flow-through adsorption experiments

A suspension containing 3 mg of adsorbent was pushed onto a 0.45 μm inorganic filter membrane using a syringe to form a thin layer of adsorbent filter membrane. About 3 mL of BF solution (15 mg L^−1^) slowly passed through the adsorbent at a rate of 4 mL min^−1^. The concentration of residual BF in solution was measured by a UV-vis spectrophotometer.

### 2.6 Adsorption experiments

The adsorption of BF was investigated in a batch system. The effect of the initial solution pH is only considered in the range of 3–8 by adding 0.1 M NaOH or 0.1 M HNO_3_ solutions because the structure of BF changes under strong acid and strong alkaline.^26^ Kinetic experiments were performed by using 60 mg of adsorbents into 60 mL of BF solution with a known initial concentration (15 mg L^−1^) at pH 6. The data were carried out under agitating magnetically at 400 rpm and at ambient temperature for 1 h. After filtration, the BF concentration in solution was measured by a UV-vis spectrophotometer (TU-1901, Peking General Instrument, China) at 543 nm based on the standard curve. The BF removal efficiency (%) was calculated according to eqn (2):

2 Removal efficiency (%) = (*C*_0_ − *C*_*t*_)/*C*_0_ × 100%

where *C*_0_ (mg L^−1^) and *C*_*t*_ (mg L^−1^) represent the concentrations of BF before and after adsorption, respectively.

Adsorption isotherms were performed with initial concentrations ranging from 10 mg L^−1^ to 75 mg L^−1^ (initial pH 6). A certain amount of adsorbents (1 mg mL^−1^) was added into the BF solution and agitated magnetically at 400 rpm. After the equilibrium of adsorption was reached, the residual concentration of BF was measured. The adsorption ability (*q*, mg g^−1^) of adsorbent against BF was evaluated using eqn (3):

3 *q*_e_ = (*C*_0_ − *C*_e_)*V*/*m*

where *C*_e_ (mg L^−1^) is the equilibrium concentration of BF, *V* (L) is the volume of the BF solution, and *m* (g) is the quantity of adsorbent.

### 2.7 Regeneration experiments

Ethanol and 0.5 M HCl solution were used to desorb BF from the adsorbent in regeneration experiments to investigate the reusability of β-CDP-COOH. The adsorbent (5 mg) was added to 5 mL of 15 mg L^−1^ BF solution at pH 6, and the mixture was stirred at 400 rpm for 1 h under room temperature. The BF removal efficiency (%) was determined by measuring the UV absorbance of supernatant solution. The adsorbent was regenerated by soaking in 0.5 M HCl and ethanol three times and then by washing with water. The regenerative adsorbent was separated by centrifugation, dried at 40 °C, and reused in another adsorption experiment. The regeneration cycle was conducted five times.

## 3. Results and discussion

### 3.1 Synthesis of β-CDP and β-CDP-COOH

**Fig. 2:**
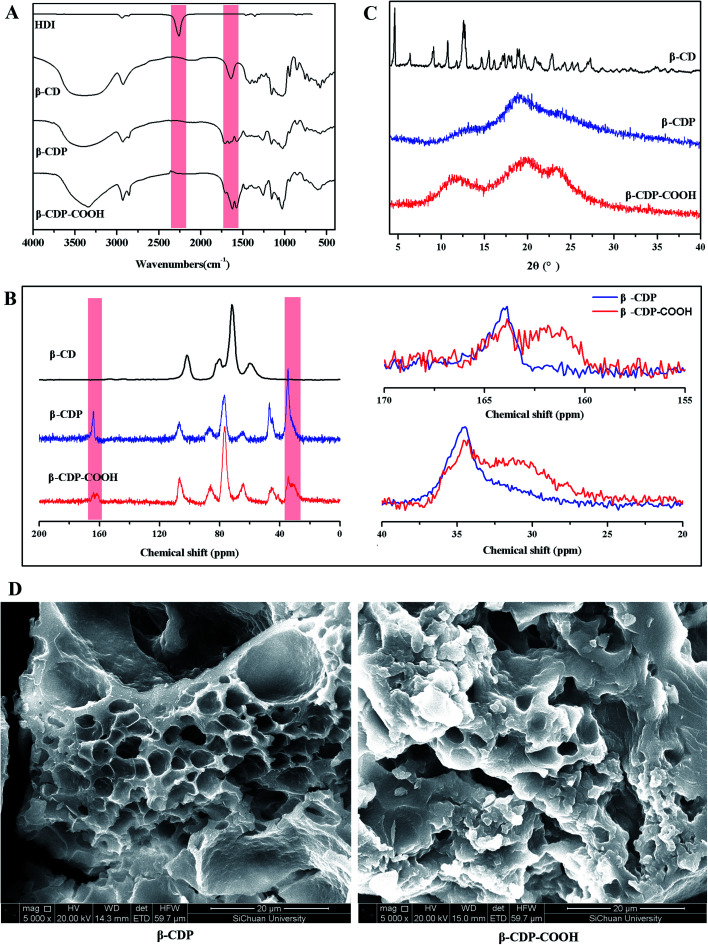
(A) FTIR spectra of HDI, β-CD, β-CDP, and β-CDP-COOH; (B) solid-state ^13^C NMR spectra, (C) PXRD patterns of β-CD, β-CDP, and β-CDP-COOH; and (D) SEM images of β-CDP and β-CDP-COOH.

The successful preparation of β-CDP and β-CDP-COOH was confirmed by FTIR and ^13^C NMR. The isocyanate groups of HDI demonstrate high reactivity with the hydroxy groups of β-CD. As shown in the FTIR spectra of β-CDP (Fig. 2A), the absorption at 2260 cm^−1^, which was attributed to the –N=C=O vibration of HDI, disappeared after the formation of the polymers. Compared with the spectrum of β-CD, new absorption peaks at 1712 and 1661 cm^−1^ emerged due to the generation of C=O of ester group and amide groups, and a new peak at 1573 cm^−1^ was assigned to N–H bending vibration. In the ^13^C NMR spectrum of β-CDP (Fig. 2B), compared with β-CD, evident new peaks were generated at 164, 46, and 34 ppm, which corresponded to the newly formed C=O and saturated carbon in the polymers. In addition, the characteristic peaks of β-CD at 101, 80, 72, and 60 ppm showed a significant shift. After the carboxyethyl group was grafted on β-CDP, the FTIR absorption peaks at 1712, 1661, and 1573 cm^−1^ shifted to 1706, 1625, and 1577 cm^−1^, respectively. This phenomenon may be due to the addition of the carboxyethyl group, which changed the electron distribution. The spectrum of ^13^C NMR β-CDP-COOH displayed new peaks at 162 and 31 ppm, which were assigned to carbons on the carboxyethyl group. Compared with β-CD and polymers, the spectra of FTIR and ^13^C NMR presented apparent changes, especially in the formation of amide groups after polymerization and the addition of carboxymethyl groups after carboxymethylation. Such phenomena indicated that β-CDP and β-CDP-COOH were successfully prepared.

### 3.2 Structural properties of β-CDP and β-CDP-COOH

Theoretically, after the cross-linking polymerization of β-CDP with HDI, it has the structural properties of an irregular network, and the structure of β-CDP-COOH obtained after carboxymethylation should be expressed as an irregular solid with negative electricity.

As shown in Fig. 2C, both β-CDP and β-CDP-COOH presented amorphous forms after polymerization and carboxylation, indicating the disorderly arrangement of β-CD molecules in polymers. SEM images (Fig. 2D) showed that the surface morphologies of β-CDP and β-CDP-COOH were inhomogeneous, which may be due to the nonuniform cross-linking reaction. According to the SEM images (Fig. S1†), both β-CDP and β-CDP-COOH have large particle sizes of about 150 μm. The surface area of β-CDP-COOH (*S*_BET_ = 9.57 m^2^ g^−1^) was slightly smaller than that of β-CDP (*S*_BET_ = 13.40 m^2^ g; Fig. S2†) probably because the graft of carboxyethyl groups occupied part of the void of β-CDP. The specific surface areas of β-CDP-COOH and β-CDP were much lower than those of the original porous materials,^18^ indicating that the porous property of the surface was not the cause of adsorption. Moreover, adsorption was attributable to the cavities of β-CD moieties and the grafted carboxymethyl groups. Furthermore, the water regain test showed that β-CD-COOH and β-CDP could retain a moderate amount of water (Table S2†), indicating that polymers are hydrophilic. The water regimen of β-CDP-COOH (approximately 116.2% of its weight) was higher than that of β-CDP (approximately 76.6% of its weight) due to the expanded net space of β-CDP-COOH by electrostatic repulsion between anions generated by the hydrolysis of carboxyl groups. The high hydrophilicity of β-CDP-COOH promoted contact with solutes in aqueous solution and facilitated the efficient adsorption of BF.

**Fig. 3:**
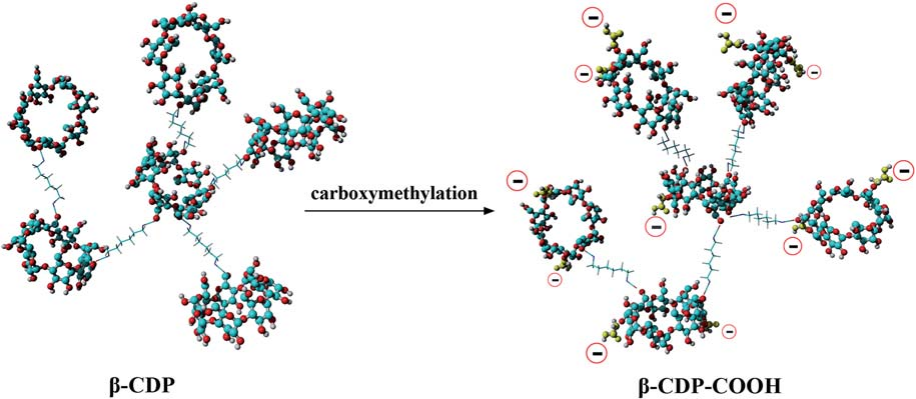
Possible structure of β-CDP and β-CDP-COOH.

Furthermore, to elucidate the effect of carboxymethyl modification on the changes in surface electrical behavior of β-CD polymers, the zeta potential was measured (Fig. S3†). β-CDP exhibited a slight negative surface charge (−4 ± 3 mV), which was due to the fact that β-CD moieties have numerous dissociable functional groups (hydroxyl groups). The zeta potential of β-CDP changed to −19 ± 0.5 mV after grafting of carboxymethyl groups. These results showed that negatively charged carboxymethyl groups were successfully introduced onto the surface of β-CDP, thereby increasing the number of active adsorption sites (Fig. 3). β-CDP-COOH exhibited higher hydrophilic ability than β-CDP, and the former's negative surface charge could drive the contact with adsorbate and provide additional adsorption sites. Thus, β-CDP-COOH produced a higher adsorption effect than β-CDP.

### 3.3 Effect of adsorption system parameters

#### 3.3.1 Initial concentrations

**Fig. 4:**
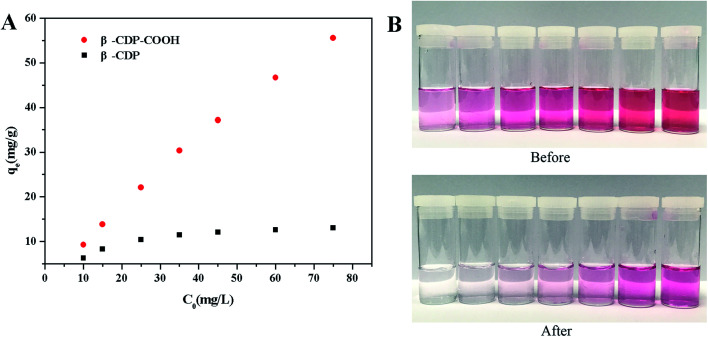
(A) Effect of initial concentration on the adsorption capacity of β-CDP-COOH and β-CDP; (B) photographs of different concentrations of BF solution before and after adsorption onto β-CDP-COOH.

The initial concentration of BF is an important driving force for the mass transfer between the aqueous and solid phases,^13^ and the removal efficiency is highly dependent on the concentration. Under the conditions of 25 °C, pH 6, solid–liquid contact time of 60 min, and initial concentration of 10–75 mg L^−1^, the removal results of BF were shown in Fig. 4. At the same concentration, a significant difference in the adsorption capacity of BF was noted between β-CDP-COOH and β-CDP. The adsorption capability of β-CDP-COOH was much higher than that of β-CDP, and it increased linearly with the increase in the BF concentration. This result may be due to the fact that the adsorption sites of β-CDP were less and soon stained by BF molecules, while β-CDP-COOH demonstrated more adsorption sites. In particular, the adsorption amount of β-CDP-COOH to BF increased with the increase of the initial concentration, while the removal efficiency decreased from 93% to 74%. Correspondingly, the adsorption amount of β-CDP remained at about 13 mg g^−1^ after a small increase, and the percentage adsorption decreased from 62% to 17%. These phenomena suggested that the removal of BF was highly dependent on the initial concentration, and the cavities of β-CD could accommodate BF with a relatively low efficiency. In addition to the cavities of β-CD themselves, β-CDP-COOH introduced a large number of carboxyl groups that could interact with the amino group of BF and form ammonium salt. Therefore, β-CDP-COOH had a higher adsorption amount than β-CDP. Given that both β-CDP and β-CDP-COOH had high removal efficiency at low BF concentration, 15 mg L^−1^ was selected as the initial concentration for subsequent studies.

#### 3.3.2 Contact time

**Fig. 5:**
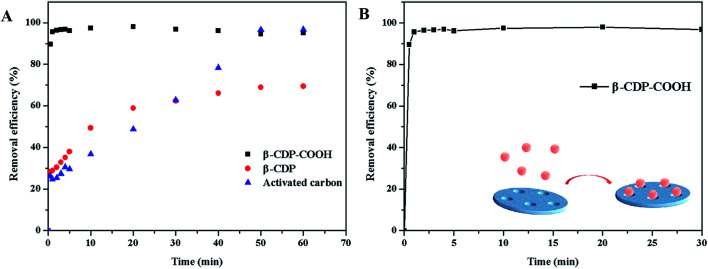
Effect of contact time on the adsorption capacity of β-CDP-COOH and β-CDP. Both (A) and (B) show the effect of contact time on the adsorption capacity. (B) shows one of the curves in (A).

The effect of contact time on the adsorption of BF was investigated at the initial concentration of 15 mg L^−1^ at 25 °C and pH 6. As shown in Fig. 5, the removal efficiency of β-CDP-COOH reached 96% within 1 min. The rapid adsorption may be due to the vacant active adsorption sites at the beginning of the adsorption process, and adsorption was completed almost instantaneously. By contrast, the adsorption curve of β-CDP increased slowly and reached equilibrium with the removal efficiency of 70% at 50 min. We also investigated the changes in the adsorption efficiency of activated carbon, a most commonly used dye adsorbent, with time. Activated carbon required 50 min to reach equilibrium with the removal efficiency of 96% and only adsorbed 25% BF at 1 min. β-CDP-COOH showed a faster adsorption than activated carbon and β-CDP, possibly because the electronegativity of β-CDP-COOH accelerated the contact rate between the adsorbent and BF. β-CDP-COOH exhibited a considerably enhanced BF removal capacity (14.4 mg g^−1^) than β-CDP (10.5 mg g^−1^). Thus, carboxymethyl modification clearly enhanced the density of active adsorption sites and boosted the BF removal capability. Compared with other β-CD-polymer reported in the literature, β-CDP-COOH also showed excellent fast adsorption ability, such as maleamic acid cross-linked β-cyclodextrin polymer reached equilibrium at 80 min,^16^ β-cyclodextrin-styrene-based polymer reached equilibrium at 90 min^17^ and β-cyclodextrin–carboxymethyl cellulose–graphene oxide composite reached equilibrium at 150 min.^18^ Therefore, β-CDP-COOH demonstrated a significant advantage over β-CDP in the removal of BF.

#### 3.3.3 Initial pH and ionic strength

**Fig. 6:**
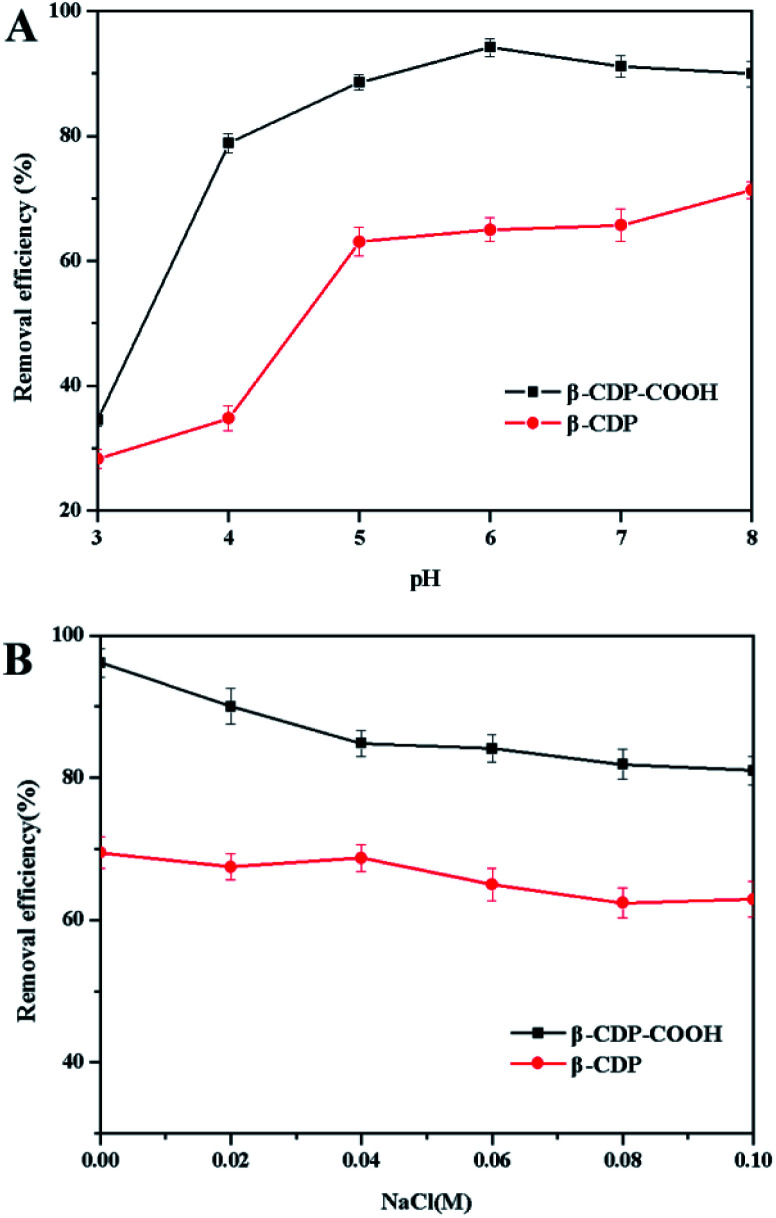
(A) Effect of initial pH and (B) effect of ionic strength on the adsorption capacity of β-CDP-COOH and β-CDP.

The initial pH of the solution plays an important role in mass transfer, which is attributed to the fact that different concentrations of hydrogen ions or hydroxide ions in the solution can change the surface charge of the adsorbent and the structure of dye molecule through the degree of dissociation.^27–29^ As shown in Fig. 6A, the removal efficiency initially changed from 35% at pH 3.0 to 80% at pH 4.0 for β-CDP-COOH. As the pH further increased from pH 4 to pH 8, the removal efficiency of BF basically remained unchanged. At pH higher than 8.0, the color of the BF solution faded, resulting in unreliable data. This phenomenon showed that at pH less than 4.0, the carboxyl groups of β-CDP-COOH retained their proton morphology (COOH) due to protonation. With the increase of pH, the carboxyl groups became deprotonated (COO^−^), and the negative charge density on the adsorbent surface increased. Thereby, interaction between the adsorbent and cationic dyes was enhanced, and amine salt formed.^7^ β-CDP showed a similar trend in the adsorption of BF. The adsorption effect improved when pH was close to neutral, and the adsorption effect was lower than that of β-CDP-COOH at any pH condition. The initial pH of the solution showed that both adsorbents exhibited high removal efficiency at pH 6.0. Therefore, pH 6.0 was selected as the initial pH of all subsequent adsorption experiments for BF.

Sodium chloride is widely used in textile dyeing process because it can promote the adsorption of textile fibers to dyes.^2^ As shown in Fig. 6B, the adsorption capacities of the polymers were affected by different concentrations of NaCl.

Addition of NaCl produced a decrease of the adsorption capacities of both β-CDP-COOH and β-CDP. This phenomenon indicated that Na^+^ would compete with BF for adsorbents surface adsorption sites. Since the surface of β-CDP-COOH has more carboxymethyl active sites, the adsorption capacity of β-CDP-COOH was more affected by Na^+^.

#### 3.3.4 Temperature

**Fig. 7:**
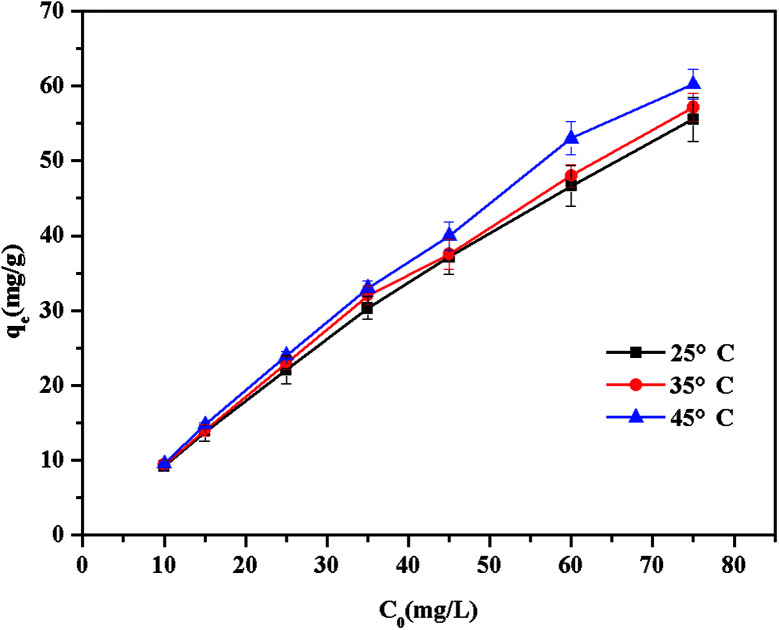
Effect of temperature on the adsorption capacity of β-CDP-COOH.

The effect of dye solution temperature on the adsorption of BF was investigated at the initial concentration of 10–75 mg L^−1^ at 25 °C, 35 °C and 45 °C. As shown in Fig. 7, with the increase of temperature from 25 °C to 45 °C, the adsorption amount of BF onto β-CDP-COOH was gradually increased, which may be due to the improvement of basic fuchsin random motion.

### 3.4 Study on the mechanism of adsorption

#### 3.4.1 Adsorption kinetics

The contact time of solid and liquid with the diffusion processes affects the rate of dye uptake.^30^ Two classical kinetic models, namely, the pseudo-first-order and pseudo-second-order models, were used to simulate the adsorption process and explore the kinetic behavior, as follows:

4 ln(*q*_e_ − *q*_*t*_) = ln *q*_e_ − *k*_1_*t*

5 *t*/*q*_*t*_ = 1/(*k*_2_*q*_e_^2^) + *t*/*q*_e_

where *k*_1_ (min^−1^) and *k*_2_ (g (mg^−1^ min)) are the rate constants of the pseudo-first-order and pseudo-second-order models, respectively; *q*_*t*_ and *q*_e_ are the adsorption amount of BF at a certain time and equilibrium time, respectively. The corresponding kinetic parameters for adsorption (Table 1) were calculated from the related liner fitting in Fig. S4.† Compared with the correlation coefficient *R*^2^, the adsorption process could be described effectively with the pseudo-second-order model for β-CDP-COOH and the pseudo-first-order model for β-CDP. The pseudo-second-order model assumes that the rate-determining step may be a chemical adsorption by sharing or exchanging electrons between adsorbate and adsorbent.^31,32^ The apparent fast rate constant (K_2_) of β-CDP-COOH for BF appeared at a relatively high level, indicating that readily accessible binding sites located at the carboxyl group of the outer surface and the interior cavities of β-CD caused the rapid adsorption of BF onto β-CD-COOH.

**Table 1:** Kinetic parameters for the adsorption of BF from aqueous solution

Kinetic models	Parameters	Adsorbent	
		β-CDP	β-CDP-COOH
Pseudo-first-order kinetic model	*q*_e_ (mg g^−1^)	5.88	0.39
	*k*_1_ (min^−1^)	0.08	0.04
	*R* ^2^	0.99	0.22
Pseudo-second-order kinetic model	*q*_e_ (mg g^−1^)	9.52	14.09
	*k*_2_ (g mg^−1^ min^−1^)	0.04	5.04
	*R* ^2^	0.97	0.99

Compared with three other β-CD-polymer, β-CDP-COOH, maleamic acid cross-linked β-cyclodextrin polymer, β-cyclodextrin-styrene-based polymer and β-cyclodextrin–carboxymethyl cellulose–graphene oxide composite had the same kinetic model, namely pseudo-second-order model.^16–18^ The results suggested that these CD polymers had the same kinetic adsorption behaviour.

#### 3.4.2 Adsorption isotherms

The adsorption isotherm, a foundation of the specific relationship between the adsorbent concentration and the adsorbent's adsorption capacity, plays an important role in the design of an adsorption system.^14^ We used four kinds of adsorption isotherms, namely, Langmuir isotherm, Freundlich isotherm, Temkin, and Dubinin–Radushkevich (D–R) isotherm models, to describe the adsorption behavior of BF onto β-CDP and β-CDP-COOH.

The Langmuir isotherm is based on the assumption that a single layer of monolayer coverage of adsorbate uniformly covers the surface of the adsorbent, and the equation is expressed as follows:

6 *C*_e_/*q*_e_ = 1/(*q*_m_*K*_L_) + *C*_e_/*q*_m_

where *q*_m_ (mg g^−1^) represent the maximum adsorption amounts of adsorption; *K*_L_ (L mg^−1^) is related to the energy of adsorption and is known as the Langmuir binding constant. *R*_L_, a dimensionless constant separation factor or equilibrium parameter, is used to predict the feasibility of an adsorption system:

7 *R*_L_ = 1/(1 + *K*_L_*C*_0_)

*R*_L_ indicates the category of the isotherm. A value greater than 1 is unfavorable, equal to 1 is linear, less than 1 is favorable, and equal to 0 is irreversible.^30^ As shown in Fig. S5,† the Langmuir model was suitable for describing the adsorption of BF onto β-CDP-COOH, and BF probably formed layers on the surface of the adsorbent due to the assumption of monolayer coverage and uniform activity distribution.^33^ Here, *R*_L_ was 0.38 for the initial concentration of 15 mg L^−1^, and this value indicated that the Langmuir isotherm was favorable for the adsorption of BF onto β-CDP-COOH. The fitting curve showed that the maximum adsorption capacity of β-CDP-COOH was 70.35 mg g^−1^. Similarly, the Langmuir model was favorable for describing the adsorption of BF onto β-CDP, and the maximum adsorption capacity of β-CDP was 13.78 mg g^−1^, which was significantly smaller than that of β-CDP-COOH. In addition, the adsorption amount of BF on β-CDP-COOH was larger than that of numerous adsorbents, such as deoiled soya (13 mg g^−1^),^1^ gangue microspheres (24 mg g^−1^),^13^ Al-MCM-41 (54 mg g^−1^),^14^ and some other β-CD-polymer reported in the literature. The maximum adsorption amounts of maleamic acid cross-linked β-cyclodextrin polymer, β-cyclodextrin-styrene-based polymer and β-cyclodextrin–carboxymethyl cellulose–graphene oxide composite were 28 mg g^−1^, 64 mg g^−1^ and 59 mg g^−1^, respectively.^16–18^

The Freundlich adsorption isotherm is an empirical equation, which assumes that adsorption on heterogeneous adsorption surfaces has different available positions and adsorption energies.^3^ The Freundlich model can be expressed as follows:

8 ln *q*_e_ = ln *K*_F_ + 1/*n* ln *C*_e_

where *K*_F_ and *n* are the Freundlich constant and surface uniformity parameter, which correspond to the degree and intensity of adsorption, respectively. The favorable range of 1/*n* is between 0 and 1.^34^ As shown in Table 2, 1/*n* for β-CDP-COOH and β-CDP were 0.49 and 0.23, so the Freundlich model was favorable for describing the adsorption of the two adsorbents. *K*_F_ of β-CDP-COOH was larger than that of β-CDP, indicating that the adsorption of BF onto β-CDP-COOH was more thorough than that onto β-CDP. The values of *R*^2^ showed that the Freundlich model was more inaccurate for β-CDP than Langmuir model. The adsorption data were analyzed by two other isotherm models, namely, Temkin and D–R isotherm models, which are expressed as follows:

9 *q*_e_ = *B* ln *K*_T_ + *B* ln *C*_e_

10 ln *q*_e_ = ln *q*_m_ − *Kε*^2^

where *K*_T_ (L mg^−1^) and *B* are the equilibrium binding constant and Temkin isotherm constant, which are related to the maximum binding energy and the heat of adsorption, respectively; *K* (mol^2^ J^−2^) is the D–R isotherm constant, and *ε* is the Polanyi potential (*ε* = *RT* ln(1 + 1/*C*_e_)). The fitting curve plots of the two isotherm models were shown in Fig. S4.† The corresponding equilibrium parameters were calculated in Table 2. The Temkin model considered the effects of indirect interactions between adsorbate and adsorbate,^35^ and the D–R model was used to distinguish physical and chemical adsorption.^36^ However, the correlation coefficient of the D–R model was 0.82, which was much lower than that of the other models, thereby suggesting that this model could not describe adsorption effectively (Table 2).

**Table 2:** Isotherm model constants and correlation coefficients for the adsorption of BF from aqueous solution

Isotherm models	Parameters	Adsorbent	
		β-CDP	β-CDP-COOH
Langmuir model	*q*_m_ (mg g^−1^)	13.78	70.35
	*K*_L_ (L mg^−1^)	0.22	0.16
	*R* ^2^	0.99	0.99
	*R*_L_	0.31	0.38
Freundlich model	*K*_F_	5.30	13.25
	*n*	4.38	2.05
	*R* ^2^	0.93	0.99
Temkin model	*K*_T_ (L mg^−1^)	4.50	2.18
	*B*	2.38	13.83
	*R* ^2^	0.97	0.97
D–R model	*q*_m_ (mg g^−1^)	12.09	48.97
	*K* (mol^2^ J^−2^)	2.28 × 10^−6^	1.50 × 10^−6^
	*R* ^2^	0.88	0.76

For all isotherm models, the Langmuir isotherm provided the most excellent fit to the adsorption data of BF on both adsorbents. This result indicated that the adsorption process is mainly monolayer adsorption and relatively homogeneous. Similarly, maleamic acid cross-linked β-cyclodextrin polymer and β-cyclodextrin-styrene-based polymer fitted the Langmuir isotherm model well, indicating that these CD polymers had the same isothermal adsorption behaviour.^16,17^

#### 3.4.3 Preliminary study on interaction mode of BF with β-CDP-COOH

**Fig. 8:**
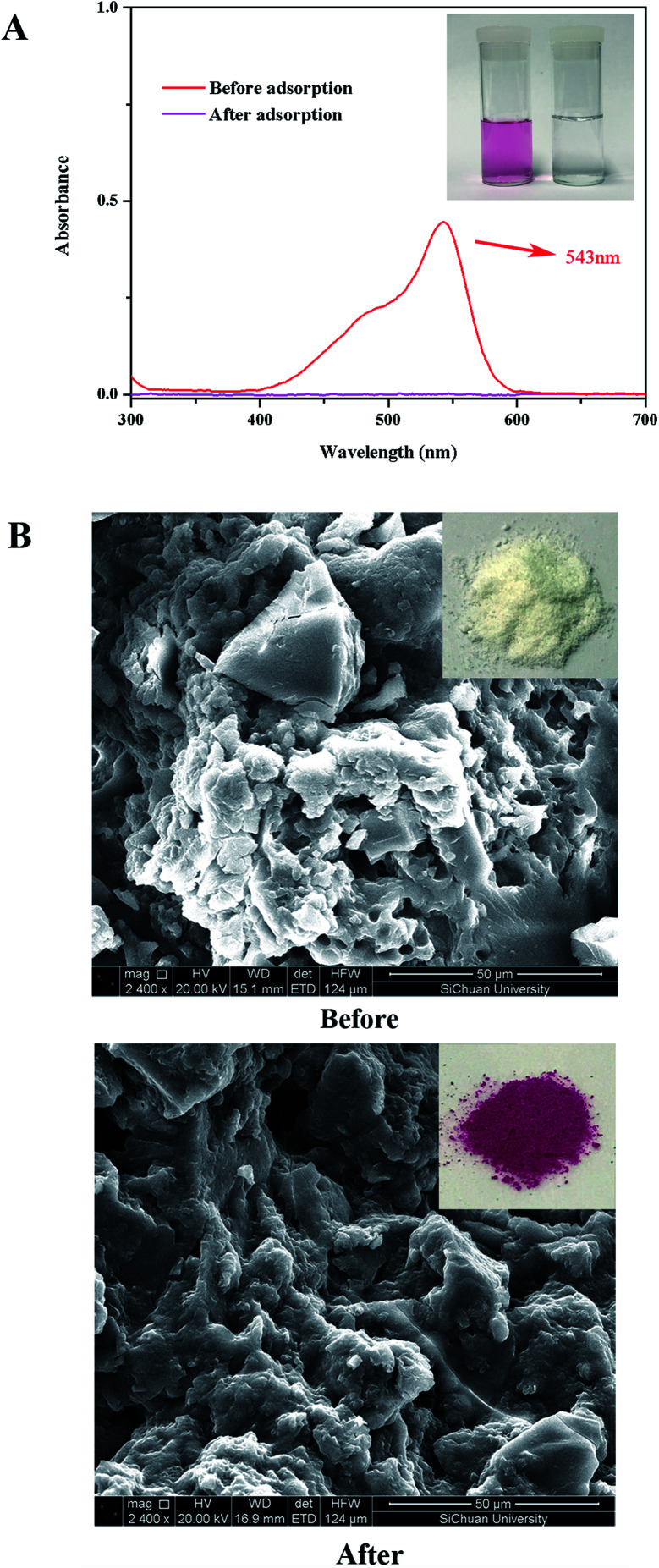
(A) UV-visible spectra of BF before and after adsorption onto β-CDP-COOH (inset: photographs of BF solution before and after adsorption); (B) SEM images of β-CDP-COOH before and after adsorption of BF.

The binding mode of BF with β-CDP-COOH was briefly investigated. Fig. 8A shows the UV-vis spectra of BF before and after adsorption. The characteristic adsorption peak of BF was greatly reduced, and the color of the BF solution changed from deep red to clean after adsorption. This change indicated that BF molecules were adsorbed on β-CDP-COOH efficiently. As shown in Fig. 8B, after the adsorption of BF, the apparent color of β-CDP-COOH changed from white to rosy red. The SEM images revealed that the morphological features of β-CDP-COOH did not change evidently before and after adsorption of BF, indicating that the structure of polymer was preserved, and the BF molecules were adhered to the surface of β-CDP-COOH.

**Fig. 9:**
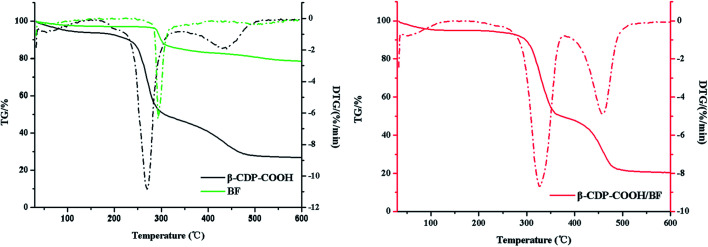
TGA spectra of BF and β-CDP-COOH before and after adsorption of BF.

When the effect of initial pH was studied, results indicated that electrostatic interaction may play a leading function in the adsorption of BF with β-CDP-COOH. To further prove the existence of the interaction between β-CDP-COOH and BF, the zeta potentials of the polymer before and after adsorption of BF were measured. After adsorption of BF, the zeta potential of β-CDP-COOH decreased from −19 ± 0.5 mV to −11 ± 1 mV. The exposed partial carboxyl group of β-CDP-COOH combined with BF cation by forming amine salts to reduce the electronegativity. Combined with the high adsorption efficiency of β-CDP-COOH, the repulsive force between the negative charges on the surface of β-CDP-COOH could expand the network pores, which is more conducive for BF molecules to enter the adsorption site. Hence, the electrostatic interaction between BF and carboxymethyl groups further accelerated contact between the adsorbent and adsorbate. In addition, thermodynamic studies demonstrated the formation of amine salts. As shown in Fig. 9, the thermal properties of β-CDP-COOH changed significantly (the fastest weightlessness at 270 °C polymer moved toward elevated temperatures) after adsorption of BF, which resulted from the high thermal stability of formed ammonium amine salts.^7^

In conclusion, BF was adsorbed on β-CDP-COOH by electrostatic attraction between ammonium ions and carboxyl groups. In addition, β-CDP-COOH retained the hydrophobic cavities of β-CD, and BF could enter the cavities of β-CD to form an inclusion complex between BF and β-CD. Therefore, the cooperative contribution of complexation and electrostatic attraction between BF and β-CDP-COOH helped improve the adsorption efficiency.

### 3.5 Desorption and regeneration

**Fig. 10:**
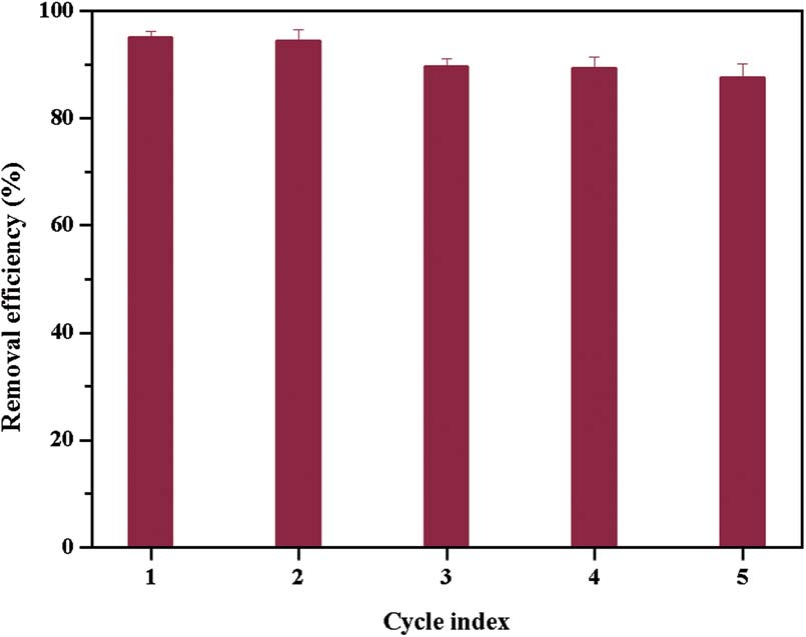
Removal efficiency of BF by β-CDP-COOH after consecutive regeneration cycles.

The reusability of adsorbents is crucial for their practical application.^37^ HCl (0.5 M) was used to regenerate the dye-adsorbed β-CDP-COOH due to low adsorption efficiency at low pH and the cation exchange process. The adsorption–desorption cycle was repeated five times, and the results were shown in Fig. 10. The removal efficiency of BF dropped from 96% to 87% after five regeneration cycles. The decline in the adsorption capacity for each subsequent cycle is a slow process because BF cannot be completely desorbed. However, the adsorption capacity of BF onto β-CDP-COOH was still an acceptable level after regenerating several times, thereby confirming that β-CD-COOH could be recycled and reused to adsorb the cationic dye BF.

### 3.6 Flow-through adsorption

**Fig. 11:**
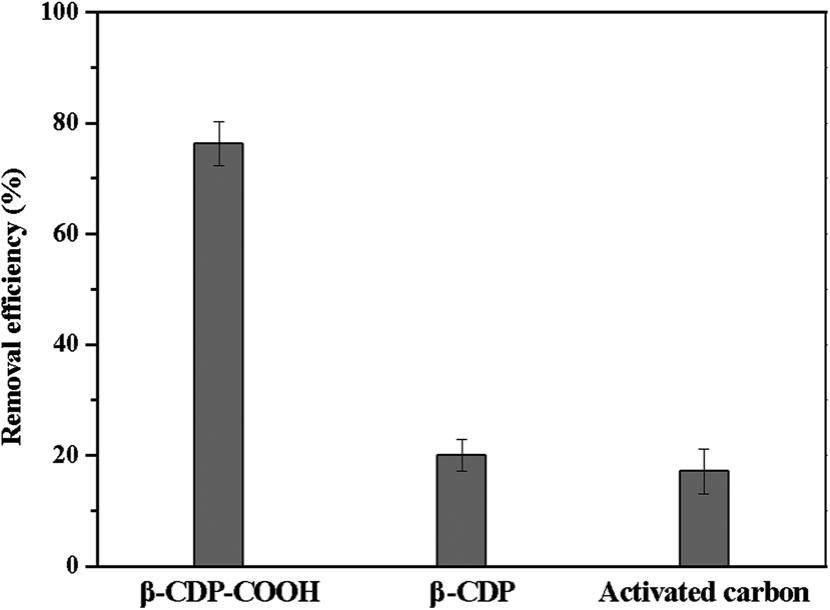
Removal of BF upon rapid flowing of the solution through a thin layer of adsorbents.

We investigated the flow-through adsorption capacity of β-CDP-COOH, β-CDP, and activated carbon at a flow rate of 4 mL min^−1^. As shown in Fig. 11, β-CDP-COOH removed 76% of BF from the solution, which was equivalent to more than 79% of its equilibrium adsorption. By contrast, β-CDP removed 20% of BF and activated carbon removed 17% of BF under the same conditions (25 °C, pH 6). Although the flow-through adsorption of β-CDP-COOH had lower removal efficiency than static adsorption due to lower solid–liquid contact efficiency, β-CDP-COOH still showed an adsorption capacity about three times larger than that of β-CDP and activated carbon. These phenomena indicated that the active sites on the surface of β-CDP-COOH could be accessed instantaneously, while more than two-thirds of the active adsorption sites of β-CDP and activated carbon were not accessible within 45 s. This result further indicated that β-CDP-COOH has a great advantage in the removal of BF.

## 4. Conclusions

This work prepared a kind of efficient irregular cross-linked β-CD polymer (β-CDP-COOH) with negative charge on the surface through a simple method. Results demonstrated that the maximum adsorption capacity (96%) of β-CDP-COOH was achieved within 1 min, while the maximum adsorption capacity (70%) of β-CDP was achieved after 50 min and the maximum adsorption capacity (96%) of activated carbon was achieved after 50 min. The high adsorption efficiency of β-CDP-COOH can be attributed to increasing the number of adsorption sites. The essence of the adsorption mechanism is that the electrostatic force provided by the carboxymethyl groups and the inclusion provided by the β-CD cavities caused BF monolayer to deposit on the adsorbent surface. The adsorption of BF onto β-CDP-COOH could be effectively described by Langmuir and pseudo-second-order models. Compared with β-CDP, β-CDP-COOH had obvious advantages in adsorption speed and adsorption capacity. The study of flow-through adsorption also provided reference for the further practical application of β-CDP-COOH. Thus, β-CDP-COOH is a potential and highly efficient adsorbent for the removal of BF or other cationic dye contaminants from wastewater.

## Conflicts of interest

There are no conflicts to declare.

## Supplementary Material

RA-010-C9RA10797E-s001
