# Supplementary material for: Preparation of a carboxymethyl β-cyclodextrin polymer and its rapid adsorption performance for basic fuchsin
Source: RSC Adv. 2020 Jun 2;10(35):20905–14. doi: 10.1039/c9ra10797e (PMC9054275; doi:10.1039/c9ra10797e)
Supplement: RA-010-C9RA10797E-s001 [file RA-010-C9RA10797E-s001.pdf]

Electronic Supplementary Material (ESI) for RSC Advances.
This journal is © The Royal Society of Chemistry 2020

Supplementary Information

Preparation of carboxymethyl β-cyclodextrin polymer and its rapid adsorption performance for basic fuchsin

Hongyu Pu, [a] Peixiao Tang, *[a] Ludan Zhao, [a] Qiaomei Sun, [a] Yuanming Zhai, [b] Zhiqiang Li, [c] Na Gan, [a] Yuanyuan Liu, [a] Xiuyun Ren, [a] and Hui Li *[a]

[a] *School of Chemical Engineering, Sichuan University, Chengdu 610065, PR China*

[b] *Analytical & Testing Center, Sichuan University, Chengdu 610064, PR China*

[c] *China Tobacco Yunnan Industrial co., LTD., Kunming 650231, PR*

* Corresponding author. Tel.: +86 028 85405220; fax: +86 028 85401207.
E-mail address: lihuilab@sina.com (H. Li); tangpeixiao@126.com (P. Tang)

Table S1. Comparison of the adsorption equilibrium time of some new adsorbents for removal BF.

Adsorbent	Water System	Adsorbent dose (g/L)	Equilibrium time (min)	q_{max} (mg/g)	References
β-CDP-COOH	triple-distilled water with pH value of 6	1	1	70	This work
bottom ash	water	4	105	7	1
deoiled soya	water	2	90	13	1
anionic polyacrylamide/graphene oxide aerogels	deionized water	0.5	4200	1034	2
cation-exchange resin	purified water	1	25	114	3
gangue microspheres	unmentioned	20	60	24	4
Al-MCM-41	unmentioned	1	90	54	5
activated carbon/ferrospinel composite	unmentioned	2	30	101	6
βcyclodextrin–carboxymethyl cellulose–graphene oxide composite	unmentioned	15	150	59	7
β-cyclodextrin-	double-	1	90	64	8

styrene-based polymer	distilled water				
maleamic acid cross-linked β-cyclodextrin polymer	purified water	3.3	80	28	9

Table S2. Water regain analysis of β-CDP and β-CDP-COOH (data were determined from the average of three measurements).

Polymers	W_d	W_w	Water regain
β-CDP	3.68	6.50	76.66%
β-CDP-COOH	3.85	8.15	111.69%

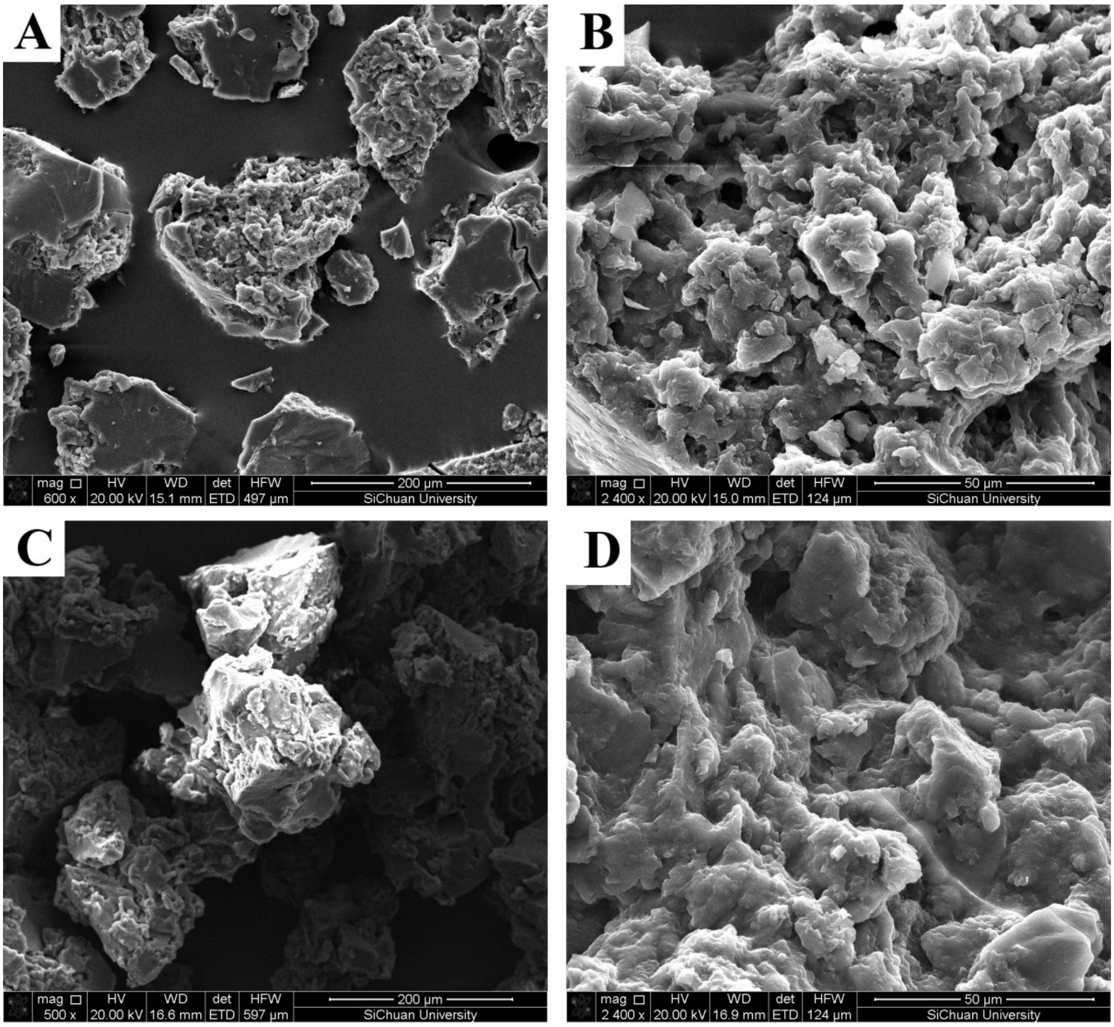

Fig. S1 SEM images of (A) β-CDP (600×), (B) β-CDP (2400×), (C) β-CDP-COOH

(500×) and (D) β-CDP-COOH (2400×)

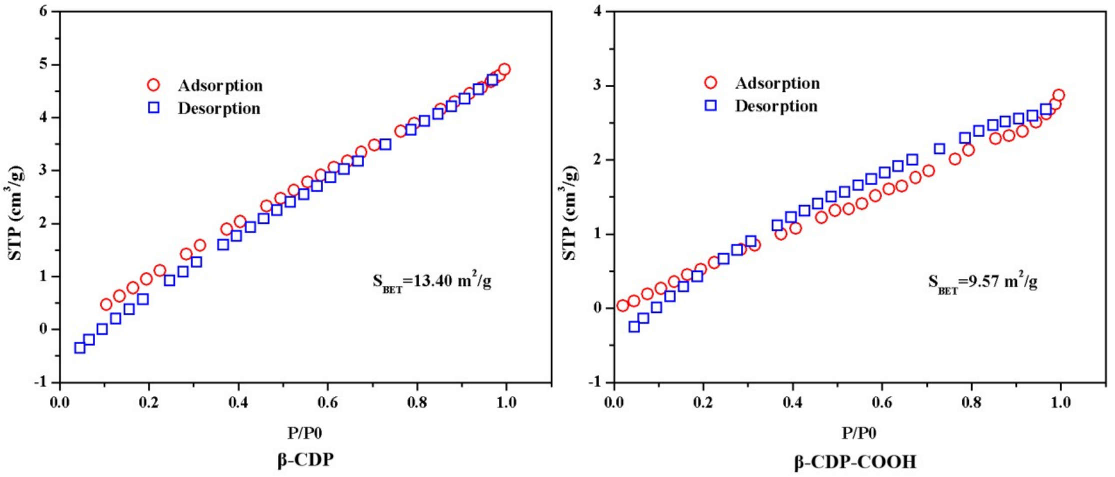

Fig. S2 N_2 adsorption−desorption isotherms and surface area data analysis of β-CDP and β-CDP-COOH.

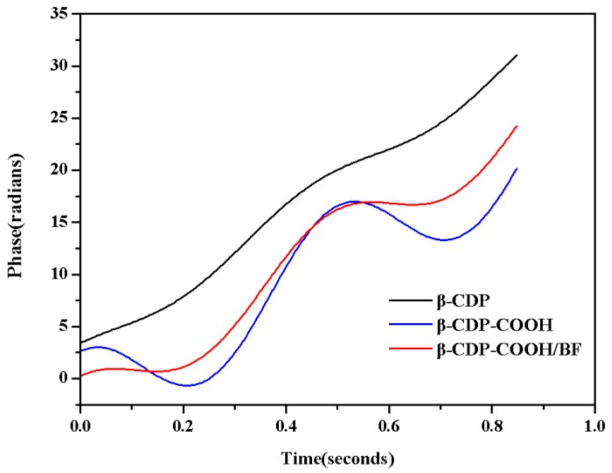

Fig. S3 Zeta potential of β-CDP, β-CDP-COOH before and after adsorption.

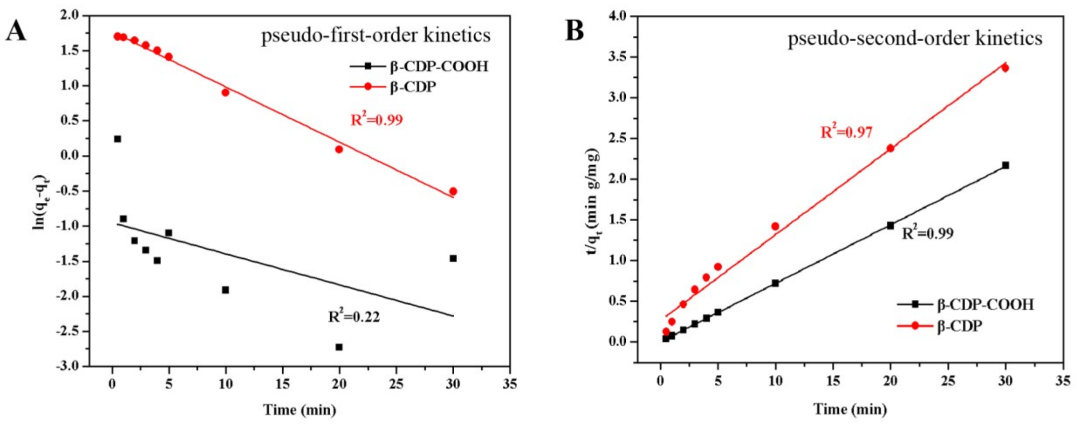

Fig. S4 Plots of (A) pseudo-first-order kinetics and (B) pseudo-second-order kinetics.

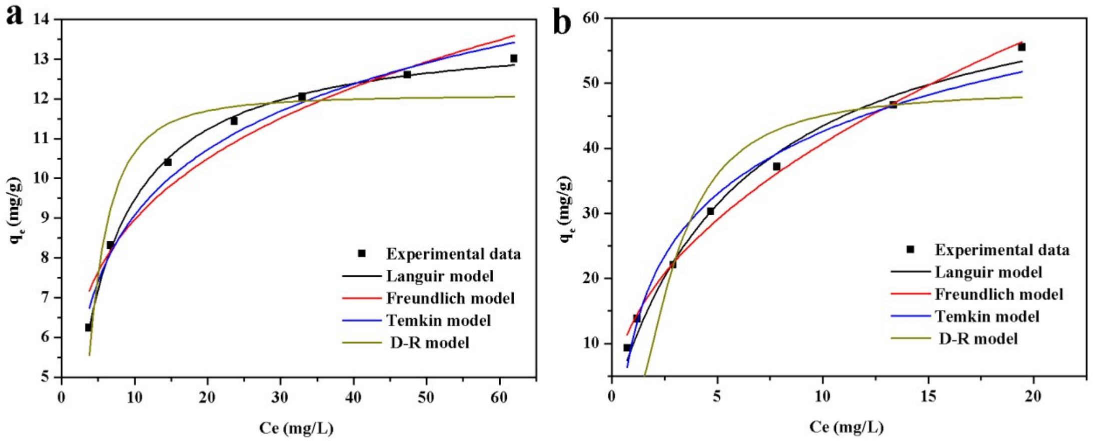

Fig. S5 Fitting curve of BF adsorption isotherms onto β-CDP (a) and β-CDP-COOH (b) of the Langmuir, Freundlich, Temkin, and Dubnin–Radushkevich (D-R) isotherm models.

References

1 V. K. Gupta, A. Mittal, V. Gajbe and J. Mittal, *J. Colloid Interf. Sci.*, 2008, **319**, 30-39.

2 X. Yang, Y. Li, Q. Du, J. Sun, L. Chen, S. Hu, Z. Wang, Y. Xia and L. Xia, *J. Colloid Interf. Sci.*, 2015, **453**, 107-114.

3 G. Bayramoglu, B. Altintas and M. Y. Arica, *Chem. Eng. J.*, 2009,**152**, 339-346.

4 S. Yan, Y. Pan, L. Wang, J. Liu, Z. Zhang, W. Huo, J. Yang and Y. Huang, *J. Adv. Ceram.*, 2018,**7**, 30-40.

5 Y. Guan, S. Wang, X. Wang, C. Sun, Y. Wang and L. Hu, *Micropor. Mesopor. Mat.*, 2018, **265**, 266-274.

6 L. Ai and J. Jiang, *Desalination*, 2010, **262**, 134-140.

7 J. Yuan, F. Qiu and P. Li, *J. Iran. Chem. Soc.*, 2017, **14**, 1827-1837.

8 X. Li, L. Xie, X. Yang and X. Nie, *RSC Adv.*, 2018, **8**, 40321-40329.

9 X. Zhang, H. Li, M. Cao and C. Chen, *Adv. Mater. Res.*, 2014, **937**, 9-16.
